# Performance assessment of public transport networks: An AHP-ANP approach

**DOI:** 10.1016/j.heliyon.2024.e40309

**Published:** 2024-11-09

**Authors:** Gang Lin, Qiuyi Zhang, Yiqun Zhang, Che Shen, Honglei Xu, Shaoli Wang

**Affiliations:** a School of Design, Fujian University of Technology, Fuzhou, 350118, China; b School of Electrical Engineering, Computing and Mathematical Sciences, Curtin University, Perth, WA 6845, Australia; c Key Laboratory of Ecology and Energy Saving Study of Dense Habitat, Ministry of Education, China; d Graduate School of Global Environmental Studies, Sophia University, Tokyo, 1028554, Japan

**Keywords:** AHP-ANP approach, Interdependencies, MCDM, Sensitivity analysis, Comparison analysis

## Abstract

Multi-criteria decision-making (MCDM) methods have been extensively employed by researchers in the public transport (PT) sector. While many studies utilize a single MCDM method, employing multiple MCDM methods can achieve more robust results. Furthermore, integrated MCDM methods have the potential to address the weaknesses and limitations inherent in single MCDM method. In this study, we address the limitations of single MCDM methods by integrating the analytic hierarchy process (AHP) with the analytic network process (ANP) to evaluate public transport networks performance. Based on the established public transport criteria matrix AHP model, we identify interdependencies among criteria. The AHP-ANP method is then applied to assess public transport networks in three case study cities in Australia. The sensitivity analysis conducted on both the AHP and integrated AHP-ANP approaches highlights the influence of criteria interdependency. This research contributes to mitigating the limitations of AHP method and applies AHP-ANP model to provide decision-makers with insights to enhance PT network performance and achieve smart PT network management.

## 1 Introduction

Public transport (PT) is a sustainable transport mode that promoting sustainable development. As highlighted by UN-Habitat in *sustainable transport, sustainable development interagency report*, promoting sustainable PT development is curial for governments to achieve *the Paris Climate Change Agreement* and *the 2030 agenda for sustainable development*. This report suggests the governments to strengthen sustainable PT transport management and planning. The development of sustainable PT entails a multifaceted process characterized by the need to balance multiple criteria. The comprehensive multiple criteria assessment of PT network provides the governments with information about future improvements. Consequently, conducting an analysis of PT network performance from diverse perspectives becomes imperative. Multi-Criteria Decision Making (MCDM) methods are frequently employed for analysing PT performance.

Despite the availability of numerous MCDM methods, no single method is universally applicable across all decision-making scenarios [1,2]. Enhanced decision-making outcomes can be achieved by employing multiple methods to tackle the same problem [3]. Such integration enables the utilization of the respective strengths of each method, while simultaneously mitigating their inherent limitations [4]. Therefore, this study systematically reviews previous research on MCDM methods in PT performance. It further integrates and develops MCDM methods to address the limitations associated with single method approaches in evaluating PT network performance.

### 1.1 Literature review

#### 1.1.1 Multiple-criteria decision-making in PT performance

The MCDM methods are the comprehensive tool that combine qualitative and quantitative aspects to evaluate complex problems and support decision-makers (DMs) in making conclusive decisions [5]. MCDM tools and applications have been used in numerous studies in the past to tackle a variety of area-specific problems, including, but not limited to, sustainability, material, environment, production management, construction and project management, energy, quality management, GIS, safety and risk management, technology and information management, manufacturing systems, operation research and soft computing, strategic management, tourism management, and supply chain management [[6], [7], [8], [9]]. The main purpose of the MCDM tools is to rank, select, sort and evaluate alternatives or criteria [6,8,9].

In the area of PT issues, MCDM methodologies are increasingly being used. According to Camargo Pérez et al. (2015), 58 different MCDM techniques have been used in the context of PT systems between 1982 and 2014, which ultimately led to the realisation that MCDM techniques have become a highly effective tool for assessing and making decisions pertaining to projects in PT systems in recent decades [10]. As a result, MCDM has emerged as a crucial decision-making method that authorities, academics, and researchers use to assess how satisfied customers are with PT systems [11].

AHP, and DEA are the two main MCDM techniques for evaluating the effectiveness of PT networks [[12], [13], [14]]. Beside AHP and DEA, there are also another four MCDM methods for evaluating and weighting PT network performance considered: PROMETHEE, TOPSIS, ANP, and ELECTRE [11,[15], [16], [17], [18], [19], [20], [21], [22]]. We arrived at the details of the six MCDM methods listed in Table 1 by referring to references related to assessing and weighting PT network performance.

**Table 1:** List of MCDM methods in evaluating and weighting PT network performance.

Reference	Specific area	Weighting method
**Cyril et al. (2019)** [22]	Assess the PT performance in quality, effectiveness, efficiency, and economic aspects.	AHP
**Sheth et al. (2007)** [23]	Measure the PT service from the users, societal, and service providers perspectives.	DEA
**Lin et al. (2023)** [21]	Measure the transit-oriented development performance degree within a zone.	ANP
**Nassereddine and Eskandari (2017)** [11]	Evaluate the PT service quality in different PT modes.	PROMETHEE
**Zhang et al. (2018)** [20]	Evaluate the PT priority implementation based on overall development level, infrastructure construction, PT service level, and policy support.	TOPSIS
**Bojković et al. (2010)** [18]	Assess transport sustainability at a macro level in terms of economic, environmental, and social aspects.	ELECTRE

However, each MCDM method has its own limitations. DEA, used to evaluate the efficiency of decision-making units (DMUs), handles multiple inputs and outputs without explicitly specifying relationships among performance criteria [[22], [23], [24], [25], [26]]. However, DEA struggles with imprecise data, assuming precise knowledge for all variables [7,26]. Most DEA application in PT consider the efficiency of PT network performance.

PROMETHEE, an outranking technique, ranks and selects among conflicting criteria but lacks a clear methodology for assigning weights, potentially resulting in negative weighting results [6,7,11,15]. PROMETHEE lacks a clear criterion weighting method.

TOPSIS identifies alternatives closest to the ideal solution, relying on Euclidean distance, which ignores attribute correlations and faces challenges in attribute weighting and maintaining judgment consistency as the number of attributes increases [7,16,20].

ELECTRE, similar to PROMETHEE, relies on decision-makers to determine criteria weights, and its outranking method impedes the direct identification of strengths and weaknesses within alternatives, complicating result and impact verification [18,19,27]. For TOPSIS and ELECTRE, the weighting process does not contain a consistency test. Therefore, these four methods are not suitable on the evaluating and weighting PT network performance context.

Due to the inputs and outputs of the MCDM methods, AHP and ANP are introduced to manage the PT criteria evaluation and ranking. Previous studies often use AHP and ANP to evaluate and weight the PT performance criteria. The details are introduced as follow.

#### 1.1.2 Analytic hierarchy process model

The AHP model is a technique for MCDM that enables DMs to deal with complex problems involving a variety of subjective and conflicting criteria [14,28]. The AHP breaks down the problem into different levels and provides a prioritised framework of choices, ranking them from most to least preferred [29]. Level objectives are established using pairwise comparisons, and weights are given to each criterion. Pairwise comparisons are used to create the factors at each level, which calls for determining the relative weights of two criteria or sub-criteria [29].

Furthermore, AHP allows DMs to handle complex issues involving multiple conflicts and subjective criteria. In terms of PT, stakeholders are concerned with both direct and indirect effects [30], and AHP addresses the financial benefit, the quality and effectiveness of the PT service, the foundational infrastructure of PT, and the degree of sustainable development. Given these areas of application, the AHP model can assist governments in more effectively monitoring and enhancing the performance of PT networks.

Despite its frequent use, AHP has been criticised for inconsistencies between criteria and ranking reversal, which can, however, be managed by testing consistency during calculations and limiting the number of criteria [7,27]. One other issue though is the need for AHP to consider setting criteria before calculation to handle interdependence among them [7].

#### 1.1.3 Analytic network process model

ANP is a more generalized model of AHP, catering to the interdependency among criteria within a hierarchical structure due to criteria interactivity [17]. In terms of merits, the model establishes a network structure where criteria, sub-criteria, and alternatives interact, allowing comprehensive communication and feedback among all network elements, and enabling interconnection between nodes (clusters) [9,17]. While ANP significantly encompasses relationships, it is not without limitations, including the necessity for exhaustive brainstorming sessions in attribute identification, the time-intensive nature of data acquisition, the higher computational requirements compared to AHP process, and the neglect of subjectivity in comparisons [31].

Although AHP and ANP can manage to evaluate and weight PT network performance criteria, ANP needs to spend more time for data acquisition and calculations. ANP is used to handle criteria interdependence, compared with AHP [7].

### 1.2 Research contribution

As discussed above, this section has presented a detailed review of current MCDM methods used in evaluating PT network performance. The current research applies singe MCDM method to attain performance reports and criteria weights. Each MCDM method contains limitations. As mentioned before, the limitation can be managed by utilizing multiple methods. Limited studies consider the interrelationship among PT network performance criteria, and the criteria hierarchy and network at the same time. To bridge this gap, this research aims to combine the MCDM methods. Hence, this paper applies AHP-ANP approach to evaluate PT network performance. Compared with mentioned research, the contribution of this paper is outlined as follows: 1. The interrelationship among PT network criteria are identified. 2. The AHP-ANP model framework is developed to assess the PT network performance. 3. The comparative analysis of the proposed method and PTCM-AHP methods are examined through three case studies. This study considers the PT network criteria using public transport criteria matrix, which identified in Lin et al. (2021) [32].

In the next section, the framework of the proposed AHP-ANP framework to evaluate PT network performance is introduced in section 2. Section 3 describes the details of case study areas. Section 4 analyses and discusses the results of three cities, and section 5 concludes the research and discusses the research limitations and future directions.

## 2 Methodology and method

**Fig. 1:**
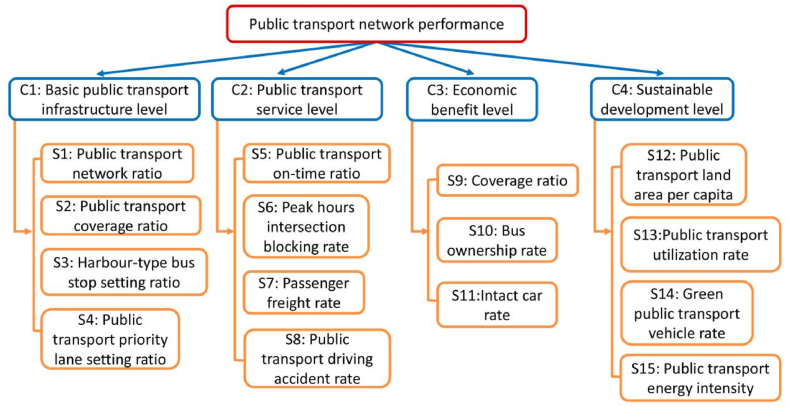
Public transport network performance criteria hierarchy structure [34].

In this paper, the PT network performance criteria hierarchy structure is established refer to Lin et al. (2021) [32]. The details of the PT criteria selection are identified in Lin et al. (2021) [32]. The evaluation framework is included 4 levels criteria which are basic PT infrastructure, PT service, economic benefit, and sustainable development [[32], [33], [34]]. The details of the subcriteria can be found in Lin et al. (2021) [32]. As can be seen from Fig. 1, the hierarchy structure involves 15 subcriteira.

**Fig. 2:**
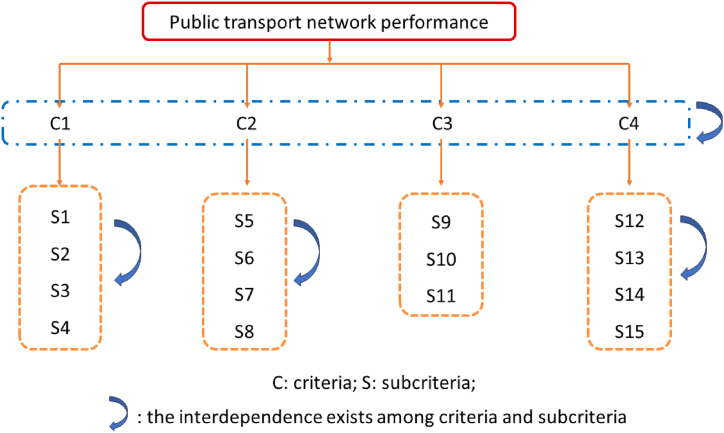
AHP-ANP model criteria structure.

By applying AHP-ANP approach, Fig. 2 shows the interdependence occurs among criteria and subcriteira. Based on the city planning policies and strategies, we identified the interdependency among the subcriteria. Refer to Fig. 2, basic PT infrastructure level is influenced by PT service level and sustainable development level criteria, and has the interdependencies exists among criteria and subcriteria. PT service level is affected by Basic PT infrastructure level criteria. Hence, the weighting results of subcriteria in basic PT infrastructure level and PT service level are calculated in ANP process. The subcriteria include PT land area per capita, peak hours intersection blocking rate, PT on-time ratio, PT priority lane setting ratio, Harbour-type bus stop setting ratio, PT coverage ratio and PT network ratio. Once the AHP-ANP model structure for evaluating PT network performance is established. And the criteria interdependency is identified. In criteria weighting process, we calculate the test the criteria weighting results. The details of weighting process are displayed as follows.

### 2.1 AHP-ANP process

**Fig. 3:**
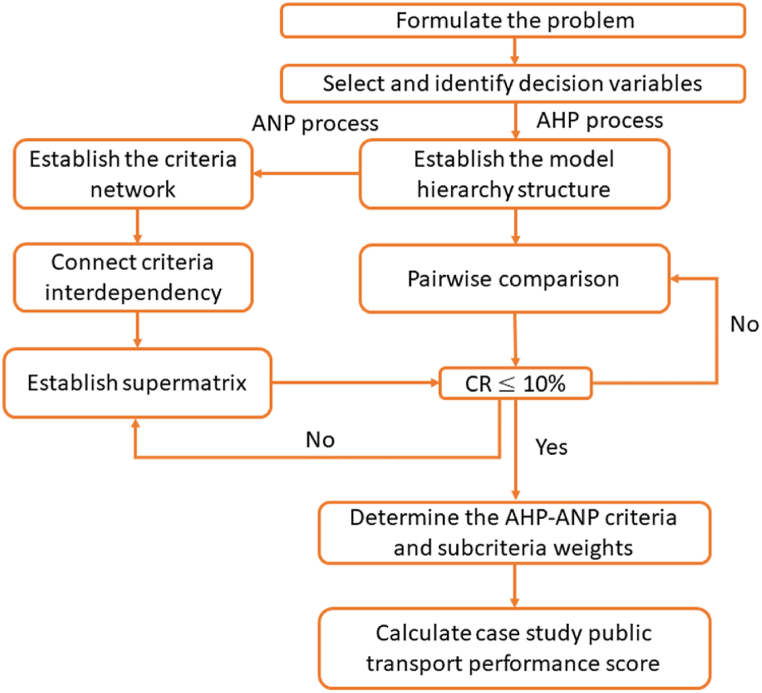
AHP-ANP model framework.

The decision variables of the proposed AHP-ANP model can be found in Lin et al. (2021) [32]. The criteria are chosen from PT evaluation indices and assessments, and the details of selected criteria are demonstrated in Lin et al. (2021) [32]. After setting the PT network evaluation model criteria and subcriteria to consider the criteria interdependence, the related subcriteria is amend the hierarchical structure into the network. As can be seen from Fig. 3, the details of the AHP-ANP model for evaluating PT network performance is contained in three processes.

Process 1 Developing AHP model structure to evaluate PT netowrk performance.

In AHP process, the AHP software is used to determine the criteria and subcriteria weights and calculates the consistency ratio for criteria hierarchy structure. There are 3 steps includes in the AHP process.

Step 1 Selecting and identifying decision variables.

As mentioned before, the selected criteria are based on existing PT performance evaluation indices and assessments [[35], [36], [37], [38], [39]]. The selected criteria details are presented in Appendix A1.

Step 2 Establishing criteria and subcriteria hierarchy AHP model structure.

Based on the selected subcriteria, the model structure is divided into 4 levels. The PT network performance criteria hierarchy structure is shown in Fig. 1 [34].

Step 3 Calculating the criteria and subcriteria weights.

Following the establishment of the hierarchical model structure for public transport network performance criteria, AHP model is utilized to assign weights to criteria and subcriteria through pairwise comparison, ranging from 1 to 9 [28]. The matrices for pairwise comparison of criteria and subcriteria are derived by reviewing the planning policies and strategies of the case study areas.

Finally, the consistency ratio (CR) for each matrix is determined. In instances where CR exceeds 10 %, it necessitates a repetition of the pairwise comparison process for criteria until CR is ≤ 10 %.

Process 2 Developing ANP model for the criteria and subcriteria.

In ANP process, the interdependencies among criteria and subcriteria are identified. Subsequently, the SupDecisions software is employed to compute the weights of the subcriteria. The process contains 3 steps.

Step 1 Identifying the interdependence exists among the criteria and subcriteria.

The interdependencies among the criteria and subcriteria, and pairwise comparison matrix are identified through a review of relevant planning policies and strategies. Saaty's 1–9 scale is utilized in the ANP framework [17]. Subsequently, the subcriteria supermatrix is employed to ascertain the weights of the subcriteria.

Step 2 Calculating the subcriteria weights.

Utilizing the SupDecisions software, the supermatrix for the supcriteria S1-S6 is established. If the CR of judgments for the supermatrix falls below 10 %, the comparison is deemed internally coherent. However, if the comparison process exhibits inconsistency, it necessitates repetition.

Step 3 Determining the AHP-ANP criteria and subcriteria weights.

Last, the criteria and subcriteria weights are determined by AHP-ANP model results. The subcritera S1-S6, and S12 wt are identified in limit super-matrix via ANP model. According to the AHP and ANP criteria and subcriteria weighting results, the model adjusts the subcriteria weights.

Process 3 Calculating case study areas PT network performance score.

Last but not least, the case study areas PT network performance score can be calculated based on AHP-ANP model criteria weights results and level grade for all subcriteria (refer to Table A1).

To validate the proposed AHP-ANP PT network performance evaluation model, the model is applied in three cities in Australia. The details of cities are demonstrated in the next section.

## 3 Case study

This study applies AHP-ANP model to evaluate the PT network performance in three Australian study areas: the City of Stonnington, the City of Bayswater, and the City of Cockburn. Furthermore, this study also conducts comparative analysis with public transport criteria matrix (PTCM)-AHP model to validate the effectiveness of the model.

**Fig. 4:**
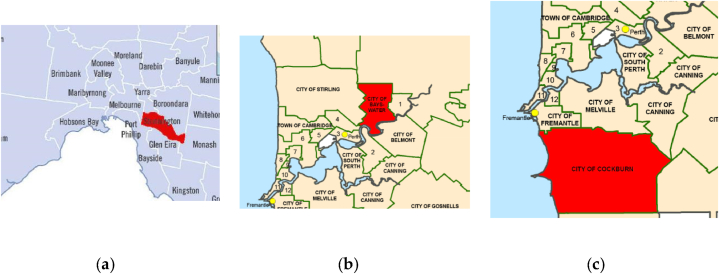
(a) City boundary of Stonnington; (b) city boundary of Bayswater; (c) city boundary of Cockburn [32].

Bayswater and Stonnington are suburbs located near the Perth and Melbourne Central Business Districts, respectively, while Cockburn is situated in the southern part of Perth. Further details on the case studies are available in Lin et al. (2021) [32]. The locations of these three cities are depicted in Fig. 4.

## 4 Results and discussion

In this section, the AHP-ANP PT network performance results are identified. To reveal the difference of criteria weighting results, the sensitive analysis between AHP-ANP model and PTCM-AHP model results is conducted.

### 4.1 AHP-ANP application

As indicated in section 3, both AHP-ANP and PTCM-AHP models contains the same criteria and subcriteria, the structure and calculation process are different. The AHP process structure and results are identified in Lin et al. (2021) [32]. The criteria weights distribution of PT criteria matrix is presented in Table B1.

In ANP process, Table 2 is the unweighted subcriteira supermatrix for subcriteria in basic PT infrastructure level, PT service level and sustainable development level. The unweighted subcriteria supermatrix is conducted by pairwise comparisons as shown in Table 2. The unweighted supermatrix is given by reviewing the current city strategies and plans. The zero value in the matrix is shown that there is no impact involved between the subcriteria.

**Table 2:** Unweighted subcriteria supermatrix.

	S1	S2	S3	S4	S5	S6	S12
**S1**	1	1	3	2	3	3	2
**S2**	1	1	3	2	2	2	2
**S3**	1/3	1/3	1	1/2	1/2	0	0
**S4**	1/2	1/2	2	1	3	0	0
**S5**	1/3	1/2	2	1/2	1	2	0
**S6**	1/3	1/3	0	0	1/2	1	0
**S12**	1/2	1/2	0	0	0	0	1

The generation of the limit supermatrix is achieved by computing the limiting power of the weighted supermatrix. Referring to Table 3, the subcriteria priorities is the weights of the seven subcriteria. The global weight of the seven subcriteria is obtained by multiplying the basic PT infrastructure level and PT service level weights.

**Table 3:** Subcriteria priorities (Limit supermatrix).

	S1	S2	S3	S4	S5	S6	S12	CR
**Weight**	25	22	7	17	10	8	11	2.11 %

### 4.2 Sensitive analysis

Based on the AHP-ANP model results, the interdependency among the subcriteria are identified, and the adjust subcriteria weights of AHP-ANP model is demonstrated in Table 4, Table 5. The global weight of criteria and ranking results of AHP-ANP and PTCM-AHP models are shown in Table 6.

**Table 4:** AHP-ANP model subcriteria weights for basic public transport infrastructure level.

Criteria	LW (%)	GW (%)	Difference (%)
**S1**	35	14.6	0.3
**S2**	31	12.7	−0.6
**S3**	10	4.1	−0.4
**S4**	24	9.8	1.9

**Table 5:** AHP-ANP model subcriteria weights for public transport service level.

Criteria	LW (%)	GW (%)	Difference (%)
**S5**	32	6.1	−0.4
**S6**	26	5	0.4
**S7**	28	5.3	–
**S8**	14	2.6	–

**Table 6:** AHP-ANP criteria Ranking results.

Criteria	AHP-ANP		PTCM-AHP	
	Ranking	GW	Ranking	GW
S1	**1**	**14.6**	**1**	**14.3**
S2	**2**	**12.7**	**1**	**14.3**
S3	**12**	**4.1**	**11**	**4.5**
S4	**3**	**9.8**	**5**	**7.9**
S5	7	6.1	7	6.5
S6	**9**	**5**	**10**	**4.6**
S7	8	5.3	8	5.3
S8	14	2.6	14	2.6
S9	**10**	**4.8**	**9**	**4.8**
S10	**11**	**4.3**	**12**	**4.3**
S11	15	1.9	15	1.9
S12	6	7.8	6	7.8
S13	13	3.2	13	3.2
S14	**4**	**9**	**3**	**9**
S15	**4**	**9**	**3**	**9**

**Fig. 5:**
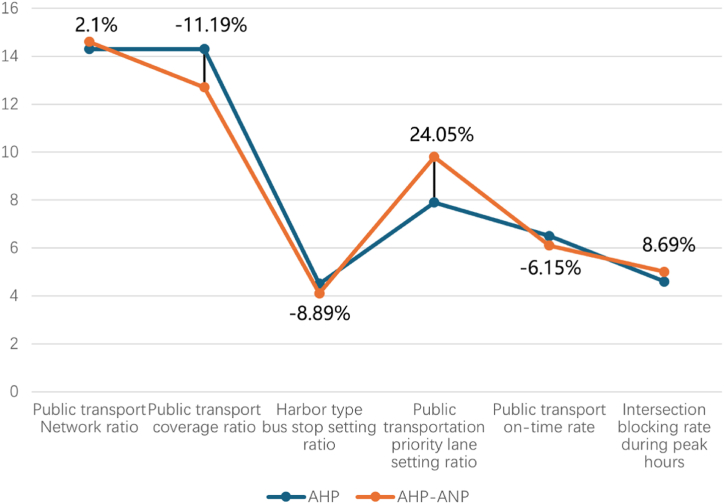
AHP and AHP/ANP subcriteria model weighting results difference rate.

Referring to Table 4, Table 6, the global weight (GW) of PT network ratio (S1) increases to 14.6 %, exhibiting a difference of 0.3 % compared to the PTCM-AHP result. Moreover, the ranking results from AHP-ANP highlight the distinction between PT coverage ratio (S2) and S1. The weight of S2 declines to 12.7 % with 11.19 % difference rate (refer to Fig. 5). Furthermore, the AHP-ANP model weight result of habor type bus stop setting ratio (S3) stands at 4.1 %, reflecting a reduction of 0.4 %. In contrast, the ranking of S4 has shifted from 5th to 3rd place. Referring to Fig. 5, this change is accompanied by a notable increase in its global weight, with a difference rate of 24.05 %.

Table 5 demonstrates that the ranking of PT on-time rate (S5) retains its 7th place, despite a decrease in weight to 6.1 % compared to the weight of S5 in PTCM-AHP model. The GW of intersection blocking rate during peak hours (S6) increases to 5 % with a growth of 0.4 %. Besides the ranking results of S1-S6 mentioned earlier, Table 6 also illustrates the differences in subcriteria ranking without any changes in GW for coverage rate (S9), bus ownership rate (S10), green PT vehicle rate (S14), and PT energy intensity (S15).

Table 7 presents the AHP-ANP model city scores for Bayswater, Cockburn, and Stonnington. The PTCM-AHP model results for these three cities are detailed in Lin et al. (2021) [32]. Bayswater's PT network achieves a score of 61.82, indicating a reduction of 2.7 % difference rate compared to the PTCM-AHP results. Cockburn's performance decreases to 65.19, with a difference rate of 2.1 %. Stonnington's city score improves to 84.43, reflecting a difference rate of 2.4 % compared to the PTCM-AHP result.

**Table 7:** AHP-ANP model case study city score.

Criteria		GW		
		Bayswater	Cockburn	Stonnington
**C1**	S1	3.09	3.37	13.25
	S2	9.34	10.27	12.7
	S3	2.7	1.51	3.19
	S4	0	0.19	8.89
**C2**	S5	5.12	5.12	4.68
	S6	0	3.16	4.72
	S7	5.3	5.3	5.3
	S8	2.01	2.01	0.76
**C3**	S9	3.34	3.34	3.86
	S10	1	1	1.06
	S11	1.9	1.9	1.9
**C4**	S12	7.8	7.8	6.62
	S13	2.99	2.99	3
	S14	9	9	9
	S15	8.23	8.23	5.5
	Total	61.82	65.19	84.43
	Difference rate	−2.7 %	−2.1 %	2.4 %

To sum up, the above discussion clearly reveals the difference on subcriteria weights obtained by AHP-ANP and PTCM-AHP model. The fluctuations in relative weights and city scores between the two models elucidate the impact of interdependencies among criteria and subcriteria on their weighting and city score outcomes. Through comparison, the integrated AHP-ANP model offers a methodology to simulate the interrelationships among PT network performance criteria. The criteria weights calculated by the AHP-ANP model reveal a higher degree of differentiation compared to the PTCM-AHP results, thereby assisting decision-makers in understanding the significance of criteria for enhancing PT network performance. Based on the AHP-ANP model results, DMs could identify the criteria and aspects that the government could consider improving in future PT plans and strategies.

## 5 Conclusion

The proposed AHP-ANP model makes significant contributions to the evaluation of PT network performance from both practical and methodological perspectives. It addresses limitations inherent in previous PTCM-AHP models by incorporating AHP-ANP frameworks to account for the interdependencies among criteria and subcriteria, thereby enabling a comprehensive assessment of PT network performance. The validity of the AHP-ANP model is demonstrated and verified through three case studies conducted in Australia. According to the model results, the model framework combines the AHP and ANP methods to obtain the robust evaluation results, which considers the criteria interdependencies and improve the effectiveness of the city PT network performance evaluation results.

The research results reveal several important findings regarding PT network performance. The AHP-ANP model highlights an increase in the global weight of the PT network ratio to 14.6 %, with a notable distinction between this and the PT coverage ratio, which saw a decrease to 12.7 %. The ranking of the public transportation priority lane setting ratio improved significantly, moving from 5th to 3rd place, accompanied by a 24.05 % rise in its global weight. Meanwhile, the PT on-time rate retained its position, although its weight decreased, and the intersection blocking rate during peak hours experienced a slight growth in weight. Additionally, no changes were observed in several subcriteria, such as PT coverage rate, bus ownership rate, and green PT vehicle rate. City performance scores revealed mixed results, with Bayswater and Cockburn showing slight declines, while Stonnington's score improved. These findings emphasize the dynamic shifts in PT network priorities and the utility of the AHP-ANP model in assessing and guiding future PT development strategies.

This novel model not only facilitates the evaluation of PT network performance within cities but also serves as a valuable tool for government entities in formulating PT network plans and strategies. The results highlight the priorities for PT network improvement, determined by the importance of criteria. Consequently, governments could implement management plans and strategies to improve PT criteria performance, guided by the criteria priorities and the respective scores.

Based on the results of this research, three case studies show the following recommendations when utilizing the AHP-ANP model. It is recommended that adjustments be made to the criteria selection and AHP-ANP structure framework to align with the specific requirements of local governments. Moreover, the incorporation of additional criteria and subcriteria warrants careful consideration, with a particular focus on reviewing the interdependencies among criteria.

Nonetheless, the limitations of this research should mitigate and manage in the future work. While we have generalized the criteria weights and ranked the PT network performance of cities based on existing local planning policies and strategies in Australia, future studies should engage experts or consider a broader selection of planning policies and strategies from diverse geographical areas to validate the research findings in a global context.

## CRediT authorship contribution statement

**Gang Lin:** Writing – original draft, Visualization, Validation, Software, Project administration, Methodology, Investigation, Formal analysis, Data curation, Conceptualization. **Qiuyi Zhang:** Writing – review & editing, Funding acquisition. **Yiqun Zhang:** Writing – review & editing, Software. **Che Shen:** Writing – review & editing, Resources, Conceptualization. **Honglei Xu:** Writing – review & editing, Supervision. **Shaoli Wang:** Writing – review & editing, Supervision.

## Data availability statement

The data presented in this study are available upon request from the corresponding author.

## Funding

This work supported by the Open Fund of the Key Laboratory of Ecology and Energy Saving Study of Dense Habitat, 10.13039/501100002338Ministry of Education, China (Grant No. 20230104).

## Declaration of competing interest

The authors declare that they have no known competing financial interests or personal relationships that could have appeared to influence the work reported in this paper.

## Figures and Tables

**Table A1:** Level grade for all sub-criteria [32].

	Level Grade	Level A	Level B	Level C	Level D	Level E
**S1 (unit: %)**	Index value interval	[60, 70]	[55, 60)	[50, 55)	[0, 50)	–
	Score interval	[90, 100]	[75, 90)	[60, 75)	[0, 60)	–
**S2 (unit: %)**	Index value interval	≥ 55	[50, 55)	[45, 50)	[35, 45)	<35
	Score interval	[90, 100]	[80, 90)	[70, 80)	[60, 70)	[0, 60)
**S3 (unit: %)**	Index value interval	[35, 100)	[25, 35)	[15, 25)	[0, 15)	–
	Score interval	[90, 100]	[75, 90)	[60, 75)	[0, 60)	–
**S4 (unit: %)**	Index value interval	≥25	[20, 25)	[15, 20)	[10, 15)	[0, 10)
	Score interval	[90, 100]	[80, 90)	[70, 80)	[60, 70)	[0, 60)
**S5 (unit: %)**	Index value interval	[95, 100]	[85, 95)	[70, 85)	[0, 70)	–
	Score interval	[90, 100]	[75, 90)	[60, 75)	[0, 60)	–
**S6 (unit: %)**	Index value interval	[0, 2]	(2, 5]	(5, 8]	(8, 11]	>11
	Score interval	[90, 100]	[80, 90)	[70, 80)	[60, 70)	[0, 60)
**S7 (unit: %)**	Index value interval	<3.5	[3.5, 4.5)	[4.5, 5.5)	[5.5, 6.5)	≥ 6.5
	Score interval	[90, 100]	[80, 90)	[70, 80)	[60, 70)	[0, 60)
**S8 (unit: times/million kilometres)**	Index value interval	[0, 1.5]	[1.5, 2)	[2, 2.5)	[2.5, 3)	>3
	Score interval	[90, 100]	[80, 90)	[70, 80)	[60, 70)	[0, 60)
**S9 (unit: %)**	Index value interval	>150	(100, 150]	= 100	[50, 100)	<50
	Score interval	[90, 100]	[80, 90)	[70, 80)	[60, 70)	[0, 60)
**S10 (unit: car/10,000)**	Index value interval	[20, 25]	[19, 20)	[18, 19)	[0, 18)	–
	Score interval	[90, 100]	[75, 90)	[60, 75)	[0, 60)	–
**S11 (unit: %)**	Index value interval	≥92	[88, 92)	[84, 88)	[80, 84)	<80
	Score interval	[90, 100]	[80, 90)	[70, 80)	[60, 70)	[0, 60)
**S12 (unit: m2/person)**	Index value interval	≥ 11	[8, 11)	[6, 8)	[4, 6)	<4
	Score interval	[90, 100]	[80, 90)	[70, 80)	[60, 70)	[0, 60)
**S13 (unit: %)**	Index value interval	[0.17, 2)	[0.14, 0.17)	[0.11, 0.14)	[0.08, 0.11)	<0.08
	Score interval	[90, 100]	[80, 90)	[70, 80)	[60, 70)	[0, 60)
**S14 (unit: %)**	Index value interval	≥95	[95, 92)	[88, 92)	[85, 88)	<85
	Score interval	[90, 100]	[80, 90)	[70, 80)	[60, 70)	[0, 60)
**S15 (unit: g standard coal/person-km)**	Index value interval	[0, 30)	[30, 80)	[80, 130)	[130, 200)	–
	Score interval	[90, 100]	[75, 90)	[60, 75)	[0, 60)	–

**Table B1:** AHP criteria weights distribution matrix [32].

Criteria	LW (%)	GW (%)
**C1: 41 %**		
**S1**	35	14.3
**S2**	35	14.3
**S3**	11	4.5
**S4**	19	7.9
**C2: 19 %**		
**S5**	34	6.5
**S6**	24	4.6
**S7**	28	5.3
**S8**	14	2.6
**C3: 11 %**		
**S9**	44	4.8
**S10**	39	4.3
**S11**	17	1.9
**C4: 29 %**		
**S12**	27	7.8
**S13**	11	3.2
**S14**	31	9
**S15**	31	9

## References

[1] Mulliner E., Malys N., Maliene V. (2016). Comparative analysis of MCDM methods for the assessment of sustainable housing affordability. Omega.

[2] Ye F., Chen Y., Li L., Li Y., Yin Y. (2022). Multi-criteria decision-making models for smart city ranking: evidence from the Pearl River Delta region, China. Cities.

[3] Lee H.C., Chang C.T. (2018). Comparative analysis of MCDM methods for ranking renewable energy sources in Taiwan. Renew. Sustain. Energy Rev..

[4] Kabir G., Sadiq R., Tesfamariam S. (2014). A review of multi-criteria decision-making methods for infrastructure management. Structure and infrastructure engineering.

[5] Khan A.U., Ali Y. (2020). Analytical hierarchy process (AHP) and analytic network process methods and their applications: a twenty year review from 2000-2019: AHP & ANP techniques and their applications: twenty years review from 2000 to 2019. International Journal of the Analytic Hierarchy Process.

[6] Behzadian M., Kazemzadeh R.B., Albadvi A., Aghdasi M. (2010). PROMETHEE: a comprehensive literature review on methodologies and applications. Eur. J. Oper. Res..

[7] Velasquez M., Hester P.T. (2013). An analysis of multi-criteria decision making methods. Int. J. Oper. Res..

[8] Mardani A., Jusoh A., Nor K., Khalifah Z., Zakwan N., Valipour A. (2015). Multiple criteria decision-making techniques and their applications–a review of the literature from 2000 to 2014. Economic research-Ekonomska istraživanja.

[9] Kheybari S., Rezaie F.M., Farazmand H. (2020). Analytic network process: an overview of applications. Appl. Math. Comput..

[10] Camargo Pérez J., Carrillo M.H., Montoya-Torres J.R. (2015). Multi-criteria approaches for urban passenger transport systems: a literature review. Ann. Oper. Res..

[11] Nassereddine M., Eskandari H. (2017). An integrated MCDM approach to evaluate public transportation systems in Tehran. Transport. Res. Pol. Pract..

[12] Barnum D.T., McNeil S., Hart J. (2007). Comparing the efficiency of public transportation subunits using data envelopment analysis. Journal of Public Transportation.

[13] Holmgren J. (2013). The efficiency of public transport operations–An evaluation using stochastic frontier analysis. Res. Transport. Econ..

[14] Boujelbene Y., Derbel A. (2015). The performance analysis of public transport operators in Tunisia using AHP method. Procedia Comput. Sci..

[15] Brans J.P., Vincke P. (1985). Note—a preference ranking organisation method: (the PROMETHEE method for multiple criteria decision-making). Manag. Sci..

[16] Olson D.L. (2004). Comparison of weights in TOPSIS models. Math. Comput. Model..

[17] Saaty T.L. (2004). Fundamentals of the analytic network process—dependence and feedback in decision-making with a single network. J. Syst. Sci. Syst. Eng..

[18] Bojković N., Anić I., Pejčić-Tarle S. (2010). One solution for cross-country transport-sustainability evaluation using a modified ELECTRE method. Ecol. Econ..

[19] Greco S., Figueira J., Ehrgott M. (2016).

[20] Zhang X., Zhang Q., Sun T., Zou Y., Chen H. (2018). Evaluation of urban public transport priority performance based on the improved TOPSIS method: a case study of Wuhan. Sustain. Cities Soc..

[21] Lin J.J., Lin T.Y., Kadali B.R., Subbarao S.S. (2023). Zone-based TOD evaluation considering interdependences among criteria and zones. Transport Pol..

[22] Cyril A., Mulangi R.H., George V. (2019). Performance optimization of public transport using integrated AHP–GP methodology. Urban Rail Transit.

[23] Sheth C., Triantis K., Teodorović D. (2007). Performance evaluation of bus routes: a provider and passenger perspective. Transport. Res. E Logist. Transport. Rev..

[24] Farrell M.J. (1957). The measurement of productive efficiency. J. Roy. Stat. Soc. Stat. Soc..

[25] Charnes A., Cooper W.W., Rhodes E. (1978). Measuring the efficiency of decision making units. Eur. J. Oper. Res..

[26] Izadikhah M., Azadi M., Toloo M., Hussain F.K. (2021). Sustainably resilient supply chains evaluation in public transport: a fuzzy chance-constrained two-stage DEA approach. Appl. Soft Comput..

[27] Konidari P., Mavrakis D. (2007). A multi-criteria evaluation method for climate change mitigation policy instruments. Energy Pol..

[28] Saaty T.L. (1994). Highlights and critical points in the theory and application of the analytic hierarchy process. Eur. J. Oper. Res..

[29] Jain S., Aggarwal P., Kumar P., Singhal S., Sharma P. (2014). Identifying public preferences using multi-criteria decision making for assessing the shift of urban commuters from private to public transport: a case study of Delhi. Transport. Res. F Traffic Psychol. Behav..

[30] Daraio C., Diana M., Di Costa F., Leporelli C., Matteucci G., Nastasi A. (2016). Efficiency and effectiveness in the urban public transport sector: a critical review with directions for future research. Eur. J. Oper. Res..

[31] Yellepeddi S., Liles D.H., Rajagopalan S. (2006). https://pomsmeetings.org/ConfProceedings/004/PAPERS/004-0069.pdf.

[32] Lin G., Wang S., Lin C., Bu L., Xu H. (2021). Evaluating performance of public transport networks by using public transport criteria matrix analytic hierarchy process models—case study of Stonnington, Bayswater, and Cockburn public transport network. Sustainability.

[33] Lin G., Xu H., Wang S., Lin C., Huang C. (2022). Performance optimisation of public transport networks using AHP-dependent multi-aspiration-level goal programming. Energies.

[34] Lin G., Xu H., Wang S., Lin C., Zhang F., Zhu J. (2024). Navigating uncertainty: a framework for optimising public transport networks' performance. Sustainability.

[35] Ministry of Transport (2014).

[36] Ministry of Construction (1995).

[37] Ministry of Housing and Urban-Rural Development (2018).

[38] Ministry of Transport (2016).

[39] Ministry of Transport (2012).

